# Slot fidelity and ligation-dependent tribology in personalized brackets made by casting versus selective laser melting: an in vitro study

**DOI:** 10.1038/s41598-025-34810-w

**Published:** 2026-01-06

**Authors:** Viet Anh Nguyen, Minh Ngoc Tran, Ngo The Minh Pham, Thi Bich Ngoc Ha, Viet Hoang, Thi Quynh Trang Vuong

**Affiliations:** 1 Faculty of Dentistry, Phenikaa University, Hanoi, Vietnam; 2 Department of Orthodontics and Pedodontics, Faculty of Dentistry, Van Lang University, Ho Chi Minh City, Vietnam; 3 Private Practice, Viet Anh Orthodontic Clinic, Hanoi, Vietnam

**Keywords:** Personalized brackets, Selective laser melting, Slot fidelity, Ligation-dependent tribology, Casting, Engineering, Materials science

## Abstract

Reliable chairside adoption of digital orthodontics depends on micrometre-scale slot fidelity and stable bracket–wire tribology. A single computer-aided design for a personalized second-premolar bracket was manufactured by lost-wax casting and by selective laser melting (SLM) (n = 36 per method). Slot height and inter-wall angle were measured on both the support-facing and non-support surfaces. Static and dynamic friction were evaluated using stainless-steel rectangular wires ligated either with tightly twisted stainless-steel ties or with elastomeric modules. Mean slot height was 480.88 ± 73.90 µm for casting and 421.47 ± 32.03 µm for SLM, against a nominal 480 µm. Overall height error did not differ between methods (*P* = 0.673), whereas angle er-ror was greater for SLM (17.76 ± 11.29°) than for casting (9.56 ± 8.88°, *P* < 0.001). The support-facing wall consistently showed reduced accuracy: in casting, height error in-creased on the support side (*P* = 0.001); in SLM, both height and angle errors increased on the support side (both *P* < 0.001). Across conditions, static friction exceeded dynamic friction (all *P* < 0.001). With steel ligation, friction was higher for casting than SLM (median static 6.00 N vs 4.35 N, *P* = 0.007; median dynamic 5.03 N vs 3.83 N, *P* = 0.011). With elastomeric ligation, the ranking reversed, with higher friction for SLM (median static 2.95 N vs 2.05 N, *P* < 0.001; median dynamic 2.54 N vs 2.03 N, *P* = 0.003). In this standardized local in vitro model, findings suggest a surface-dependent reduction in accuracy at the support-facing wall and a fabrication-by-ligation interaction that may alter the frictional ranking between steel and elastomeric ligations. Cast brackets showed more consistent full-wire seating, whereas SLM brackets may benefit from calibrated design offsets and targeted finishing to limit undersized or tapered slots; validation beyond this setup is needed.

**Supplementary Information:**

The online version contains supplementary material available at 10.1038/s41598-025-34810-w.

## Introduction

Digital dentistry is shifting orthodontic appliance design from catalog selection to data-driven personalization. Intraoral scanning and computer-aided design (CAD) workflows now integrate appliance planning, allowing brackets to be designed around patient-specific crown morphology and intended biomechanics rather than generic tooth forms^1^. Within this ecosystem, additive manufacturing, particularly selective laser melting (SLM), offers geometric freedom for complex appliances and integrated features, while conventional routes such as lost-wax casting from 3D-printed sacrificial patterns remain widely used and clinically familiar^2,3^. The clinical performance of any bracket nevertheless hinges on micron-scale slot fidelity and tribology at the bracket-wire-ligation interface, where small geometric or surface deviations can materially alter engagement, play, and resistance to sliding^4–6^.

Most prior evaluations of slot fidelity and friction have focused on stock, off-the-shelf brackets manufactured by metal injection molding or computer numerical control, with many reports of oversized slots and non-parallel walls under ISO-based measurements^6–9^. Frictional behavior is likewise brand- and ligation-dependent under standardized wires, with consistent differences among steel ties, elastomeric modules, and self-ligating designs^10,11^. By contrast, comparatively few studies have assessed personalized brackets, and fewer still have contrasted metal fabrication routes for the same digital design, including the influence of build-side surfaces inherent to layerwise processes^12,13^. Emerging work on personalized SLM brackets demonstrates that, when process parameters are optimized, high geometric precision can be achieved^14^.

The present in-vitro study addresses this gap by comparing personalized brackets produced by lost-wax casting from 3D-printed patterns and by SLM from a single CAD design. The a priori hypotheses were that casting and SLM would not differ in slot fidelity and frictional behavior. Based on the results, it is intended to provide practical guidance for orthodontists and technicians on selecting fabrication routes to achieve reliable slot fidelity and predictable frictional performance in personalized brackets.

## Methods

### Study design

This investigation employed a two-group comparative design, contrasting brackets manufactured by casting from a 3D-printed sacrificial pattern with those manufactured by selective laser melting. Reporting adhered to the modified CONSORT checklist for in-vitro studies of dental materials^15^. A priori power analysis was performed for a two-sided comparison between methods with an alpha level of 0.05 and a power of 0.9. Based on slot size values reported by Park et al. for cast brackets (0.02299 ± 0.00056 inch) and computer-aided manufacturing brackets (0.02257 ± 0.00092 inch), the minimum required sample size was calculated to be 35 brackets per group^9^. In the present study, 36 brackets per group were analyzed to ensure adequate power.

### Fabrication of personalized brackets

A second-premolar personalized bracket was digitally designed from a premolar crown in the Autolign library (version 1.6, Diorco, Gyeonggi-do, Korea). The nominal slot height was 480 µm (0.0189 inch), the nominal slot depth was 660 µm (0.0260 inch); and the mesiodistal slot length was 2.00 mm (0.079 inch). This single digital design was used to fabricate 72 brackets in total (n = 36 per fabrication method).

Cast brackets were manufactured in a nickel–chromium alloy using the lost-wax technique. Sacrificial patterns were printed with a liquid–crystal-display (LCD) printer (Saturn 2, Elegoo, Shenzhen, China) using a castable photopolymer resin (Dental Castable, Elegoo). Printing parameters included a layer thickness of 50 µm, a build angle of 90°, and the lateral bracket surface oriented downward. A standardized support strategy was employed. A master support (1.0-mm diameter) was positioned at the center of the lateral surface, and auxiliary supports (tip diameter 0.4 mm; base diameter 0.6 mm) were distributed to scaffold the full height of the slot wall. After printing, auxiliary supports were removed, and a wax runner was joined to the master support to sprue the pattern. Wax was deliberately not applied within the slot to prevent ingress. The invested pattern was then burned out, and the metal was cast.

**Fig. 1:**
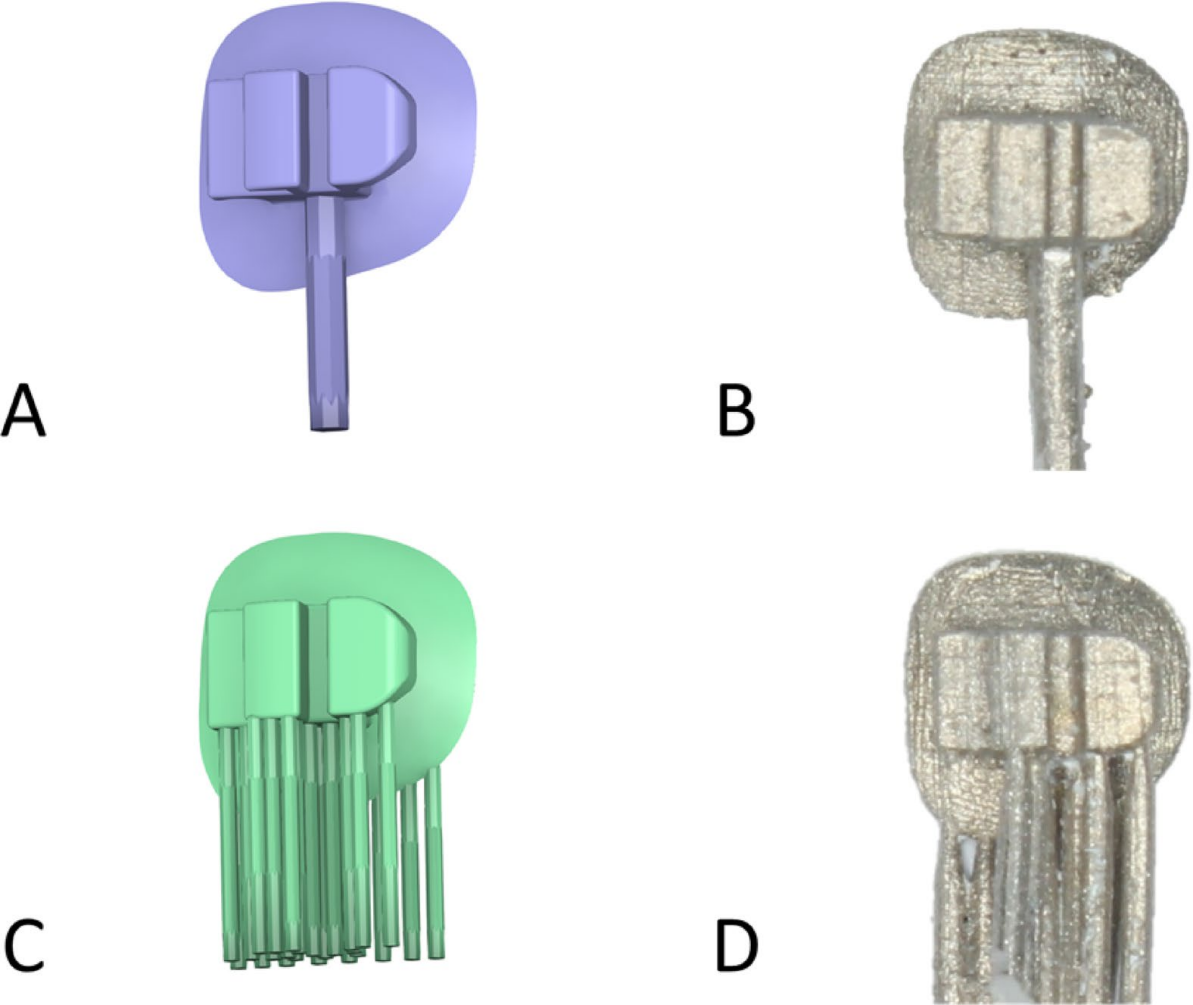
Digital design of personalized brackets. (**A**, **B**) Lost-wax casting pattern 3D printed in photopolymer resin with a single master support, auxiliary supports removed. (**C**, **D**) Selective laser melting built with support struts.

SLM brackets were fabricated from cobalt–chromium alloy powder using the DeskFab X1 system (FastForm 3D Technology, Yancheng, China). The build angle and orientation matched the casting protocol (90°, lateral surface downward). Support struts (0.4 mm diameter) were applied to the lateral surface to maintain continuous support of the slot wall during building (Fig. 1A–D). SLM processing parameters included: single CW fiber laser, 300 W; laser spot size 60 µm; scanning-head speed 950 mm/s; scanning step 50 µm; layer-thickness 50 µm; upper-feeding, one-way recoating. Post-build stress-relief heat treatment was subsequently performed following the alloy vendor’s cobalt–chromium dental recommendation.

After fabrication, all metallic supports were removed under magnification with careful deburring of the lateral surface that previously carried supports. Brackets from both groups underwent identical finishing by rotary tumbling (KT-6808, Tasanol, Shenzhen, China) at 300 rpm for 72 h to standardize deburring and to homogenize near-surface asperities without targeted polishing of the slot interior. Rotary tumbling was carried out in an aqueous polishing slurry containing mixed media: microbeads (0.3–1.0 mm diameter) and stainless-steel cylindrical pins (0.4 mm diameter × 4 mm length). No additional directed finishing steps (e.g., grit blasting, electropolishing, or paste-polishing) were intentionally performed on the slot walls; therefore, any smoothing of the slot surface occurred only as an indirect consequence of tumbling and was expected to be similar across groups. This approach reduces operator-dependent variability while preserving manufacturing-route signals in the slot region.

### Dimensional measurements

**Fig. 2:**
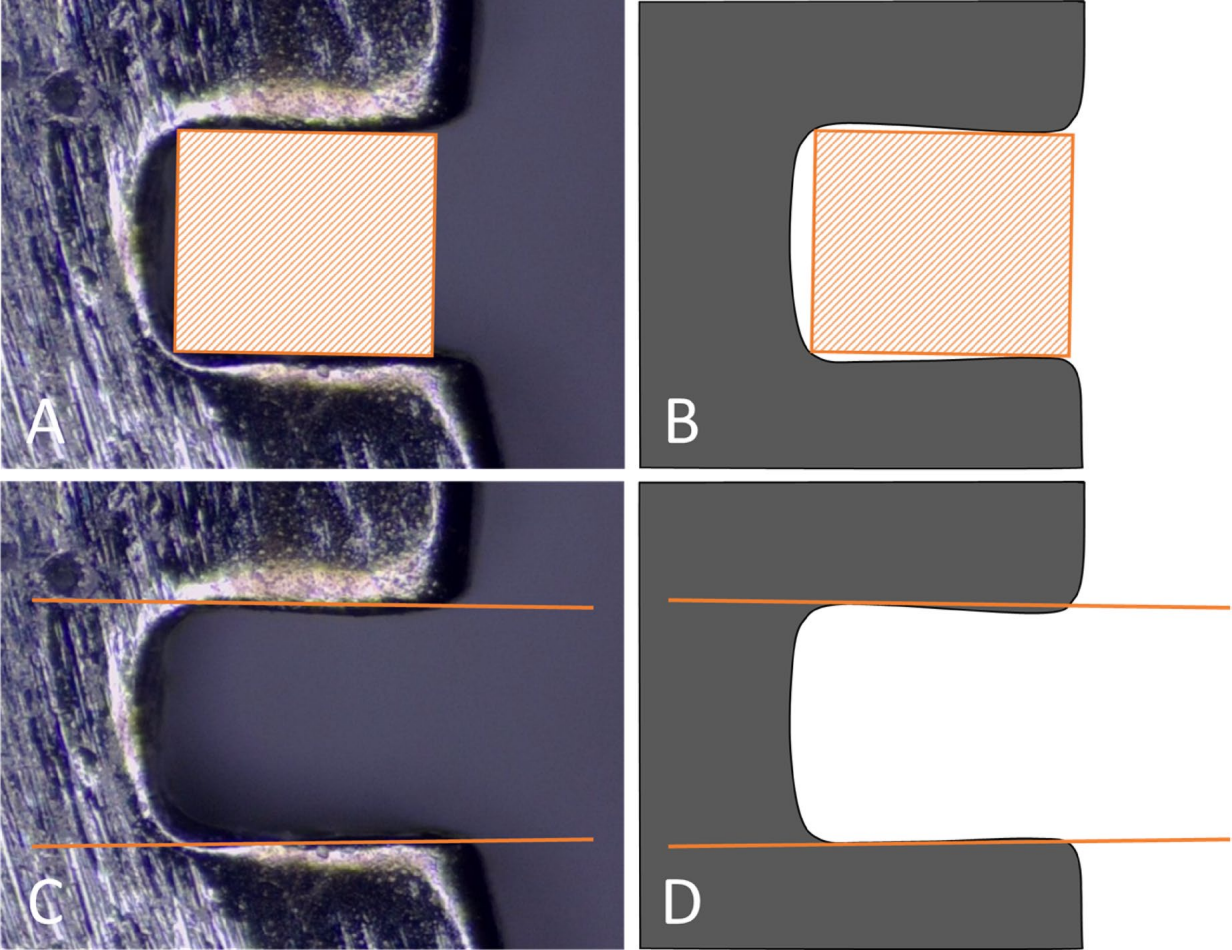
Dimensional measurements of bracket slots under a digital stereo microscope. (**A**, **B**) Slot height is determined as the principal dimension for archwire engagement. (**C**, **D**) Inter-wall angle is measured as the angle between the two opposing slot walls.

Slot height and slot-wall parallelism were assessed using a digital stereo microscope (XL45B3, Tiger, Guangzhou, China). To ensure a consistent measurement angle during slot metrology, each bracket was seated in a custom 3D-printed measurement jig that located the bracket on three datum surfaces, orienting the slot plane perpendicular to the optical axis and the slot long axis horizontal. This measurement jig was used only for dimensional measurements; friction experiments employed a separate tooth-analog jig. The jig was mounted on the microscope’s goniometric stage; yaw and pitch were set to 0° and verified by simultaneous focus of both slot walls at a fixed working distance and magnification. Pixel size was calibrated with a stage micrometer. Images were analyzed with ImageJ software (National Institutes of Health, Bethesda, MD, USA). Slot height was selected as the principal dimension, as it determines archwire engagement: a reduced height prevents archwire insertion, whereas an increased height introduces play and compromises torsional control. The inter-wall angle, defined as the angle between the two opposing slot walls, was measured using a Pissa Ruler virtual protractor (IO Stream, Ho Chi Minh, Vietnam)^16^. All dimensional measurements were performed in accordance with ISO 27,020 guidelines for orthodontic brackets^7^. The effective slot size was determined as the largest rectangular gauge that could be inserted into the slot without deformation, operationalizing the slot as a precisely fitting rectangular box (Fig. 2A–D). Measurements were performed separately on the surface that had been supported during additive manufacturing and on the unsupported surface.

Among bracket slot dimensions, height was prespecified as the primary dimensional outcome because third-order clearance (“torque play”) is governed by the clearance in the occluso-gingival direction together with the archwire’s true height and edge-bevel geometry. By contrast, depth does not enter torque-play models and mainly influences seating as a function of ligation force, whereas length is orders of magnitude much larger than manufacturing deviations (20–50 µm) and has no effect on torque^17^. Furthermore, prior studies on bracket slot fidelity prespecified slot height as the primary dimensional outcome^7,18,19^. Accordingly, we analyzed height and slot-wall parallelism as outcomes and report depth and length as nominal design values to avoid terminological ambiguity.

### Friction testing

**Fig. 3:**
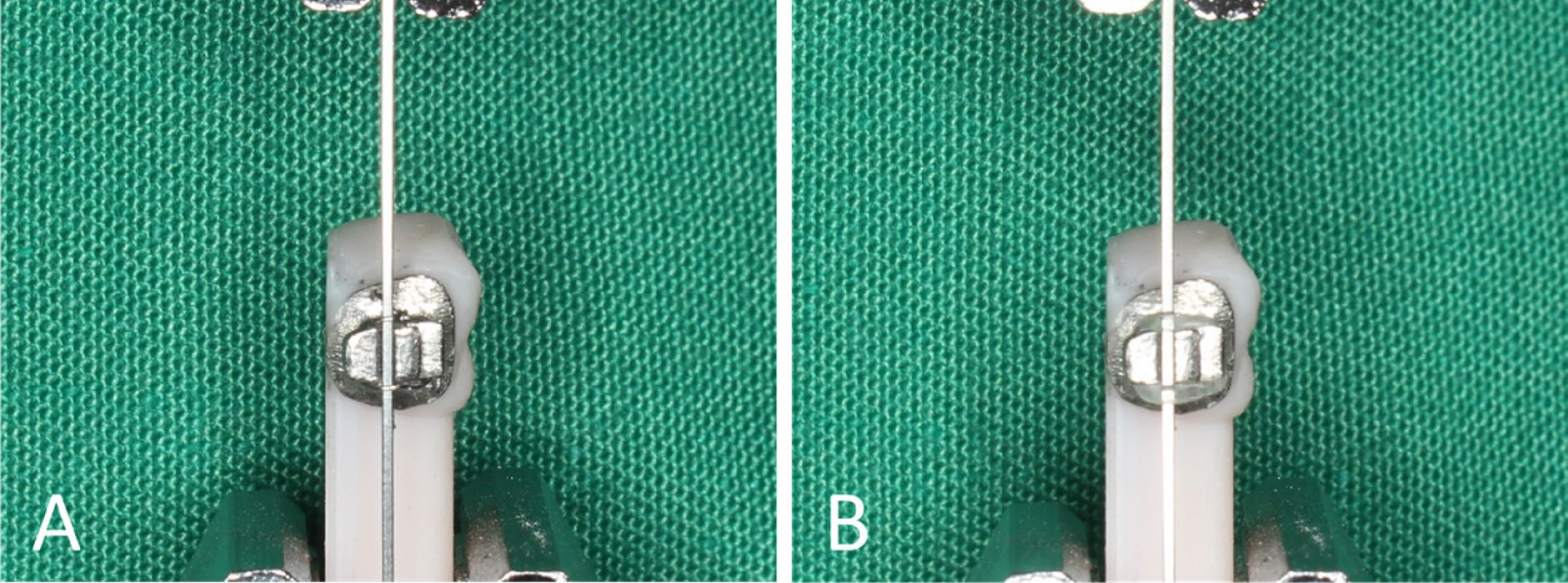
Test setup showing the vertical wire in the upper clamp and the bonded bracket on the tooth-analog jig in the lower clamp. Before each run, the grips were adjusted to align the archwire with the line of pull, and the wire was verified to be straight and passive. (**A**) Steel ligature. (**B**) Elastic ligature.

A customized tooth-analog jig was designed in Meshmixer (Autodesk, San Rafael, CA, USA). The jig incorporated a horizontally oriented premolar crown and a vertical fixture to ensure that, after bonding, the bracket slot was aligned vertically. Segments measuring 30 mm of 0.017 × 0.025-inch stainless-steel archwire (3B, Hangzhou, China) were ligated to the slot in two ways, with tightly twisted stainless-steel ligatures to fully seat the wire and with elastomeric modules (3B) (Fig. 3A, B). For each run, a new wire segment, a new bracket, and new ligatures were used to eliminate wear- or deformation-related carryover. The bracket–wire assembly was mounted in a universal testing machine (HP-1 kN, Handpi Instruments, Shenzhen, China). The tooth-analog jig was rigidly clamped to the lower grip so that the bracket was 10 mm above the lower grip face. The upper grip clamped the archwire 10 mm above the bracket, and a 10 mm wire tail extended below the bracket. Before each run, the grips were adjusted so that the wire coincided with the machine’s line of pull and passed centrally through the bracket slot without lateral contact. The alignment was checked visually from the frontal and lateral views, and the grips were re-tightened only after the wire remained straight and passive (no bending or pre-tension), maintaining zero angulation at the start of each run.

Static friction was defined as the peak force required to initiate sliding from rest, whereas dynamic friction was defined as the mean force measured over a 5-mm travel distance. During friction testing, artificial saliva (Salisol, QD-Pharma, Hanoi, Vietnam) was applied with a calibrated micropipette to fully wet the slot–wire interface (50 µL immediately before each run), thereby standardizing the lubrication condition across runs. This approach represents an initial-wetting in vitro condition rather than continuous salivary flow. The crosshead speed was 1 mm/min. All forces were recorded in newtons (N).

### Statistical analysis

All variables were tested for normality using the Shapiro–Wilk test, and none followed a normal distribution; therefore, nonparametric tests were applied throughout. To evaluate measurement reliability, a second examiner repeated the full set of slot-size, angle, and friction tests. Inter-rater agreement was quantified using a 2-way random-effects, absolute-agreement intraclass correlation coefficient for single measurements (ICC(2,1)), complemented by the Dahlberg method error and Bland–Altman analysis. Descriptive statistics are reported as median with interquartile range in addition to mean ± standard deviation. Within-method comparisons of bracket surfaces (support vs no-support) for slot height and angulation errors were performed with the Wilcoxon signed-rank test. Between-method comparisons for each surface and for the averaged values were assessed with the Mann–Whitney U test. Similarly, for friction experiments, static and dynamic conditions within each ligature type were compared using the Wilcoxon signed-rank test, and cast versus SLM groups were compared using the Mann–Whitney U test. All statistical tests were two-sided with a significance threshold of 0.05. Effect sizes were reported alongside nonparametric tests. For paired comparisons (support versus no-support; static versus dynamic), we computed the rank-biserial correlation (r_rb_)​. For independent two-sample contrasts (cast versus SLM), we reported Cliff’s δ. 95% confidence intervals were obtained via bootstrap resampling (2,000 iterations). Analyses were conducted in Python (version 3.4; Python Software Foundation, Wilmington, DE, USA).

## Results

**Fig. 4:**
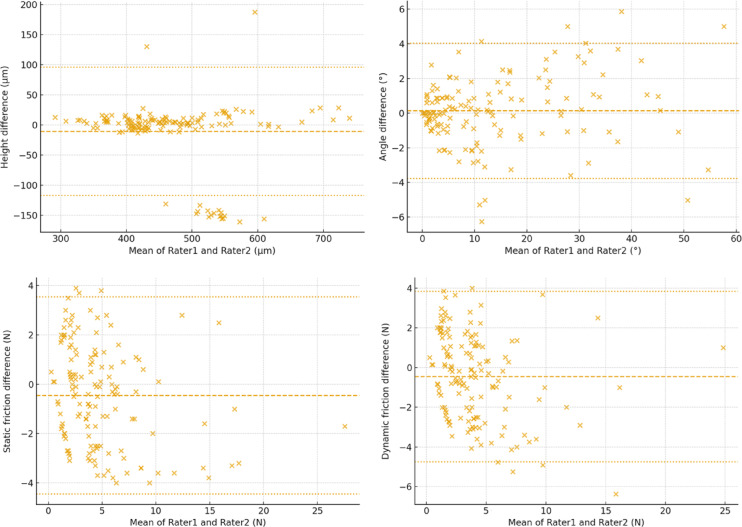
Bland–Altman plots of inter-rater agreement for bracket slot height, angle parallelism, static friction, and dynamic friction.

Inter-rater agreement was high overall (Table 1). Angle parallelism showed excellent reliability with ICC (2,1) = 0.99 and trivial bias (0.1°), whereas bracket slot height demonstrated good reliability with ICC = 0.81 and a small negative bias (− 10.8 µm). For friction, reliability was good for static (ICC = 0.87) and moderate-to-good for dynamic (ICC = 0.81); Bland–Altman plots showed small negative mean differences (− 4.5 N and − 4.6 N, respectively) with clinically acceptable limits of agreement (Fig. 4).

**Table 1:** Inter-rater reliability of bracket slot height, angle parallelism, and frictional resistance measurements.

Measure	n (pairs)	ICC	95% CI	Method error	Bias	SD of difference	Limit of agreement
Bracket height	144	0.812	0.715–0.886	39.074	− 10.788	54.384	− 117.381–95.804
Parallel angle	144	0.989	0.984–0.992	1.407	0.131	1.992	− 3.774–4.036
Static friction	144	0.873	0.785–0.917	14.744	− 4.549	20.419	− 44.570–35.473
Dynamic friction	144	0.806	0.657–0.878	15.771	− 4.571	21.907	− 47.509–38.366

**Fig. 5:**
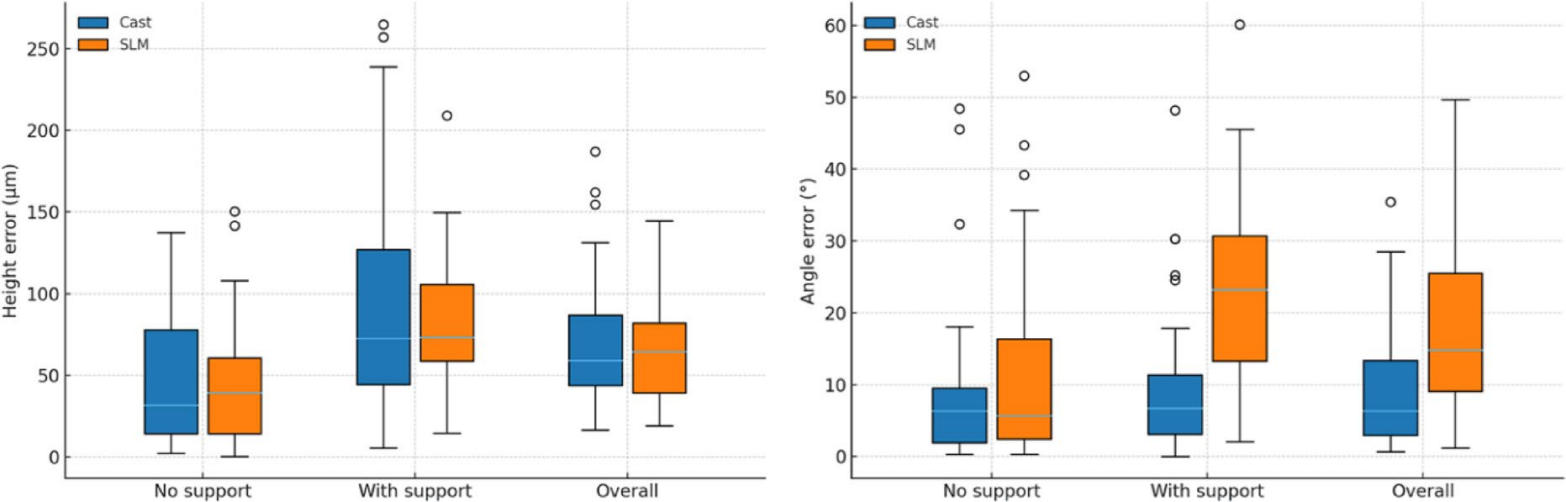
Box-and-whisker plots of height (µm) and angle errors (°) for cast and SLM brackets at the no-support, with-support, and overall surfaces.

Relative to the software-specified nominal design value of 480 µm, the cast group’s average slot height (480.88 ± 73.90 µm) was closer to the target, whereas the SLM group undershot (421.47 ± 32.03 µm). In the cast group, height error on the support surface was greater than on the no-support surface (*P* = 0.001), whereas angle error did not differ between surfaces (*P* = 0.307) (Table 2 and Fig. 5). In the SLM group, both height and angle errors were higher on the support surface than on the no-support surface (both *P* < 0.001). Between fabrication methods, height error did not differ at the no-support surface (*P* = 0.960), the support surface (*P* = 0.822), or overall (*P* = 0.673). By contrast, angle error was similar at the no-support surface (*P* = 0.454) but was higher for SLM at the support surface (*P* < 0.001) and overall (*P* < 0.001).

**Table 2:** Comparison of height and angle slot errors between cast and SLM brackets, measured at no-support, support, and overall average surfaces.

	Height error (µm)				Angle error (°)			
Group	No support	With support	Overall	P^1^ r_rb_ (95% CI)	No support	With support	Overall	P^1^ r_rb_​ (95% CI)
Cast	31.86 (14.44–77.85) 44.64 ± 37.81	72.77 (44.51–126.94) 97.15 ± 74.86	59.01 (44.01–87.02) 70.90 ± 42.11	0.001 0.60 (0.30–0.85)	6.39 (1.95–9.57) 9.03 ± 11.41	6.75 (3.16–11.39) 10.08 ± 10.37	6.38 (3.02–13.41) 9.56 ± 8.88	0.307 0.20 (− 0.17–0.58)
SLM	39.30 (14.44–60.68) 45.08 ± 37.37	73.39 (58.82–105.93) 79.30 ± 41.39	64.71 (39.42–82.22) 62.19 ± 29.93	< 0.001 0.68 (0.40–0.89)	5.69 (2.47–16.43) 12.44 ± 14.45	23.26 (13.32–30.75) 23.07 ± 13.02	14.86 (9.11–25.59) 17.76 ± 11.29	< 0.001 0.70 (0.41–0.92)
P^2^ Cliff’s δ (95% CI)	0.969 − 0.01 (− 0.28–0.27)	0.826 0.03 (− 0.25–0.30)	0.681 0.06 (− 0.21–0.33)		0.454 − 0.10 (− 0.38–0.17)	< 0.001 − 0.62 (− 0.82– − 0.40)	< 0.001 − 0.50 (− 0.72– − 0.26)	

CI, confidence interval; r_rb,_ rank-biserial correlation; SLM, selective laser melting; Values are presented as median (interquartile range), and mean ± standard deviation; P^1^ Significance level for comparison between no-support and support surfaces within the same fabrication method (Wilcoxon signed-rank test); P^2^ Significance level for comparison between cast and SLM groups for the same parameter (Mann–Whitney U test).

**Fig. 6:**
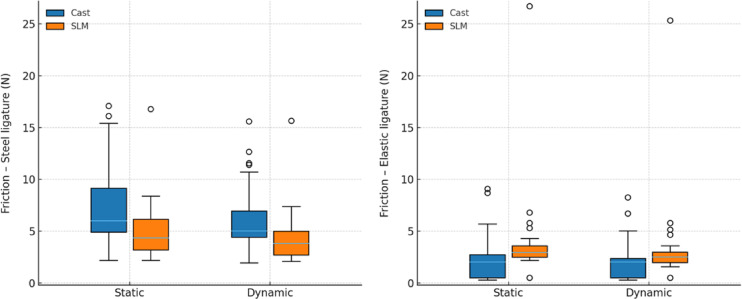
Box-and-whisker plots of friction with steel and elastic ligatures for cast and SLM brackets under static and dynamic conditions.

In both cast and SLM groups, static friction was significantly higher than dynamic friction for steel and elastic ligatures (all *P* < 0.001) (Table 3 and Fig. 6). When comparing fabrication methods, cast brackets exhibited greater friction with steel ligatures under both static (*P* = 0.007) and dynamic (*P* = 0.011) conditions. By contrast, SLM brackets showed higher friction with elastic ligatures in both static (*P* < 0.001) and dynamic (*P* = 0.003) conditions.

**Table 3:** Friction with steel and elastic ligatures for cast and SLM brackets under static and dynamic conditions.

	Steel ligature			Elastic ligature		
Group	Static friction (N)	Dynamic friction (N)	P^1^ r_rb_​ (95% CI)	Static friction (N)	Dynamic friction (N)	P^1^ r_rb_ (95% CI)
Cast	6.00 (4.90–9.15) 7.34 ± 4.12	5.03 (4.42–6.96) 5.93 ± 3.19	< 0.001 − 1.00 (− 1.00–1.00)	2.05 (0.50–2.75) 2.13 ± 2.20	2.03 (0.50–2.39) 1.89 ± 1.85	< 0.001 − 1.00 (− 1.00–1.00)
SLM	4.35 (3.20–6.15) 5.00 ± 2.70	3.83 (2.71–5.01) 4.33 ± 2.44	< 0.001 − 1.00 (− 1.00–1.00)	2.95 (2.50–3.60) 3.83 ± 4.08	2.54 (1.99–2.98) 3.23 ± 3.93	< 0.001 − 1.00 (− 1.00–1.00)
P^2^ Cliff’s δ (95% CI)	0.007 0.37 (0.12–0.62)	0.011 0.35 (0.10–0.61)		< 0.001 − 0.53 (− 0.74– − 0.28)	0.003 − 0.40 (− 0.63– − 0.14)	

CI, confidence interval; r_rb,_ rank-biserial correlation; SLM, selective laser melting; Values are presented as median, interquartile range, and mean ± standard deviation. P^1^ Significance level for within-method comparison of static versus dynamic friction (Wilcoxon signed-rank test); P^2^ Significance level for between-method comparison for the same condition (Mann–Whitney U test).

## Discussion

This study compared slot fidelity and friction of personalized metal brackets fabricated by lost wax casting and selective laser melting, evaluating support facing versus non-support surfaces and steel versus elastomeric ligation. Based on the results, two a priori hypotheses were assessed. First, SLM would not differ from casting in slot fidelity. This was not supported, as height deviations were broadly comparable, while SLM exhibited different angle errors at the support surface and overall. Second, casting and SLM would exhibit different frictional behavior. This was supported, with cast brackets showing higher friction under steel ligation and SLM brackets showing higher friction under elastomeric ligation, indicating a bracket-ligation interaction.

Regarding fabrication choices, the casting arm employed an LCD printer with castable resin to fabricate burnout patterns, as this material is specifically engineered for clean burnout with negligible ash under standard investment schedules and yields well-defined margins suitable for dental casting. LCD exposes an entire layer through a masked light engine, giving uniform exposure and fine in-plane detail with broadly available, low-cost hardware in dental labs^20^. Alternative patterning such as fused deposition modeling leaves pronounced layer lines and is less reliable for fine dental features and burnout; while high-end PolyJet and digital-light-processing castable systems can achieve comparable quality; however, their costs are substantially higher and they rely on proprietary consumables^21–23^. For direct metal fabrication, we selected laser powder-bed fusion (SLM) because it achieves high density and accurate fit for dental cobalt–chromium components and has been widely validated in prosthodontic applications.^24–26^ We did not use binder jetting because post-print sintering introduces substantial shrinkage and residual porosity that compromise dimensional fidelity; we avoided directed-energy deposition because its larger bead size and heat-affected zone limit the resolution required for narrow bracket slots; and we avoided electron-beam melting due to its vacuum requirement and typically rougher as-built surfaces^27–29^. Looking ahead, a hybrid route—printing the bracket body additively and precision-milling the slot—could combine geometric freedom with tight slot tolerances^30^.

We note that the casting pathway used a nickel–chromium alloy whereas the SLM pathway used a cobalt-chromium alloy. Differences in hardness, work-hardening, and native oxide chemistry between nickel–chromium and cobalt-chromium could influence both near-surface topography after identical finishing and the friction coefficient at the bracket–wire interface. Accordingly, part of the between-group friction contrast may reflect alloy effects rather than fabrication route alone. Future designs should control for this by casting and SLM with the same alloy family where feasible, and reporting alloy-resolved microhardness and roughness data to deconvolute material from process effects.

Compared with SLM, cast brackets showed larger slot heights. The group mean was close to the nominal value, but the dispersion was substantially wider. This pattern is consistent with the expansion of the investment during setting and burnout, partly offset by alloy shrinkage during solidification. When these opposing effects do not balance, the mold cavity and the resulting slot become larger than nominal, a behavior repeatedly reported for cast orthodontic brackets ^16^. The wider deviations in the cast group likely accumulate across multiple steps, including inaccuracies in the printed wax pattern, investment handling, and the casting process itself. In contrast, SLM produced near-net-shape parts, yet small internal apertures tended to be undersized relative to the digital design. Contributing mechanisms include finite melt-pool and beam width with insufficient contour offset encroaching on the feature, heat buildup in narrow geometries that broadens the melt pool, and down-skin roughness with adhered or partially melted powder that narrows the effective opening^31,32^. Consequently, subnominal slot heights in SLM are expected and reflect melt-pool physics, thermal history, and powder adhesion rather than isolated surface artifacts. Appropriate contour and hole offsets, orientation to avoid critical overhangs, and tuned scan parameters can restore dimensions toward nominal^33,34^.

Notably, the cast group’s near-nominal mean contrasts with much of the literature, which typically reports oversized slots and divergent walls in metal brackets^6,8,9^. However, our finding of undersized SLM slots closely parallels Yang et al., who observed smaller-than-nominal slot heights in as-printed personalized SLM brackets^14^. On the other hand, although Brucculeri et al. reported remarkably precise slot height for in-house 3D-printed resin brackets, those data pertain to photopolymer resin appliances rather than metal and should not be generalized to SLM metal brackets^35^.

Across both fabrication routes, the support-side surface consistently exhibited greater dimensional error than the non-support surface. This trend reflects differences in how each surface is constrained during layerwise formation. The non-support surface is continuously underlaid by previously solidified material, which stabilizes the geometry throughout the building. By contrast, the support-side surface is borne by discrete struts that leave short unsupported spans between contact points. These spans are more susceptible to local heat accumulation, micro-sagging, and post-processing disturbances during support removal and finishing, which cumulatively degrade fidelity. In the casting pathway, errors on the support side were expressed primarily as height deviations of the slot, whereas inter-wall angle remained comparable between surfaces. Imprints of support features and their removal on the printed pattern could be transferred to the investment and then amplified by investment expansion and alloy shrinkage during solidification. These effects altered the slot opening in the height direction but might not impose a systematic moment that would tilt the slot walls. In the SLM pathway, the disadvantages on the support side were more pronounced. At this downward-facing surface, residual irregularities from the build and subsequent support removal tended to produce a taper through the slot height, which in turn increased the inter-wall angle error.

Across all tests, static friction exceeded dynamic friction, consistent with classic sliding mechanics^36–38^. The higher friction we observed with stainless-steel ligatures versus elastomeric modules is most plausibly due to greater seating force. Tightly tied steel ligatures press the archwire more firmly into the slot, increasing resistance to both breakaway and sliding. Prior work shows that friction scales with ligation force and that changing the ligation mode, such as loose versus tight steel ties or or conventional elastomerics versus specialized low-friction elastomeric modules, can invert the steel-elastic hierarchy depending on tie force^11,39,40^. For example, Hain et al. reported that saliva-lubricated “slick” elastomeric modules reduced static friction by up to 60% versus standard modules, and that loosely tied stainless-steel ligatures generated the least friction^39^.

Ligation-specific patterns align with our geometry and surface findings. Under steel ligation, friction was higher for cast than SLM in both static and dynamic modes. With the strong seating imposed by steel ties, the archwire is driven firmly into the slot base and one wall. In cast brackets, the combination of near-nominal slot height and broader within-group dispersion likely increased real contact and breakaway resistance. Under elastomeric ligation, the trend reversed, with SLM exceeding cast for both static and dynamic friction. Because elastomeric ligatures apply lower normal force than tight steel ties, friction becomes more sensitive to slot clearance and to surface features at the support-facing wall. In our data, slightly undersized SLM slots and support-facing irregularities promote earlier binding, whereas slightly larger cast slots preserve play and reduce sliding resistance. These effects are consistent with greater plowing and adhesive contributions during sliding under low normal force^41^. Therefore, two practical takeaways follow: when steel ligatures are planned for sliding mechanics, SLM brackets may offer lower resistance; where elastomeric modules are preferred, casting may be advantageous.

Tribological behavior was quantified in sliding tests; however, because internal-wall roughness was not directly measured, our mechanistic interpretation emphasizes geometric fidelity and ligation force. We did not perform surface profilometry because, at the slot scale, neither contact stylus probes nor optical profilometry can reliably access or resolve the internal slot walls. Narrow apertures and steep sidewalls cause tip interference, shadowing, and focus loss, while contact probing risks altering the surface; critically, the friction-determining roughness resides on these internal walls. Nonetheless, known differences in near-surface topography between cast nickel–chromium and SLM cobalt-chromium—such as partially sintered particles and down-skin facets in SLM, or investment-derived micro-pits in casting—could modulate the adhesive and ploughing components of friction, especially under low normal forces with elastomeric ligation. Quantifying roughness and bearing-area curves on support-facing versus non-support walls will be important to separate clearance-driven binding from topography-driven resistance in future studies.

Clinically, archwires are changed throughout treatment; however, we restricted friction testing to a single 0.017 × 0.025-inch stainless-steel rectangular wire because it is the workhorse during space closure—the phase most sensitive to friction. More flexible nickel–titanium wires are typically avoided at this stage because their lower stiffness can accentuate bowing in sliding mechanics, whereas beta-titanium may be used selectively for torque and formability, but generally exhibits higher friction than stainless steel, making it less favored for low-friction space closure^42^. Conversely, larger rectangular wires (0.018 × 0.025-inch ) are typically reserved for full torque expression; in sliding mechanics they generate excessive friction and are therefore not routinely used for space closure. This rationale guided our choice to fix the wire type and size so as to isolate fabrication-route effects. Follow-up studies should include other archwire materials, round and rectangular sections in other sizes, clinically relevant edge-bevel radii, and progressive wire sequences to map fabrication-ligation–wire interactions across typical treatment stages.

The observed manufacturing-mechanics relationship has practical implications for bracket design. Although SLM reproduced the intended overall bracket form, the slot dimensions deviated systematically from nominal, with undersized height and larger angular error at the support-facing surface. In this condition, full archwire engagement is not guaranteed, and torque expression may be compromised^5^. The apparently lower static friction of SLM under some conditions likely reflects incomplete seating of the archwire rather than genuinely reduced resistance. In contrast, cast brackets averaged closer to the nominal dimension and allowed consistent engagement, though with wider variability and higher friction under steel ligation. Taken together, these findings suggest that current SLM workflows may not yet be suitable for fabricating very small and sensitive components such as bracket slots, where micron-level fidelity is critical for both archwire engagement and biomechanical efficiency. Future improvements should focus on compensating CAD offsets and process parameters to recover nominal slot height and wall parallelism, targeted post-processing of the support-facing wall, and hybrid routes in which the bracket base and body are additively manufactured and the slot is finished by precision milling^43,44^.

This in-vitro study used a single bracket design, one wire size and alloy, one build orientation per method, and one finishing protocol, which limits generalizability across geometries, materials, and surface treatments. Friction was measured in a simplified straight-line setup with artificial saliva and a fixed crosshead speed, without superimposed tip, torque, or bracket malalignment that occur clinically. Saliva was applied as an initial-wetting bolus rather than continuously perfused. Slot dimensions were derived from 2D optical measurements and gauge insertion rather than full 3D metrology. While 2D optical measurements and virtual protractors comply with ISO-based slot-dimension assessments, they cannot capture full 3D slot geometry, including torsional skew, local waviness, or the cumulative effect of micro-undercuts. Likewise, optical height/angle measurements do not quantify surface roughness parameters, which can contribute to ploughing and adhesion during sliding. Ligation force was not standardized by direct measurement, and only the immediate condition was assessed. Steel ligatures were evaluated at baseline without mechanical or thermal aging, so time-dependent changes such as progressive loosening, wire-ligature fretting, or metal fatigue were not captured. Elastomeric force decay over time was likewise not modeled. Results should therefore be interpreted as initial performance under controlled conditions, and future work should include force-calibrated ligation, long-term aging, multiple wire alloys and sizes, varied orientations and finishing routes, roughness metrics, and 3D geometry characterization (micro-CT or profilometry).

## Conclusions

Within the strict local conditions of this in vitro protocol, personalized brackets from a single CAD design showed surface-orientation-dependent dimensional fidelity and a fabrication-by-ligation interaction in sliding resistance. The support-facing wall tended to be the less accurate surface in both casting and SLM. Slot-height errors were of similar magnitude between methods, whereas SLM tended to show greater slot-angle deviation driven mainly by the support-facing side and a tendency toward undersized slots. Static values were generally higher than dynamic values across conditions. With tightly twisted steel ligatures, sliding resistance was higher for casting than for SLM; with elastomeric modules, the ranking reversed, suggesting that clearance and surface condition may dominate under lighter seating forces. Accordingly, SLM brackets should not be presumed to enhance torque control or reduce resistance unless slot geometry is compensated toward nominal. Future work should evaluate calibrated CAD offsets and process parameters, targeted finishing of the support-facing wall, and hybrid workflows in which the body is additively manufactured and the slot is precision-milled. Prospective in vivo validation under saliva, thermal cycling, biofilm, and ligature force decay is warranted.

## Supplementary Information

Below is the link to the electronic supplementary material.

Supplementary Material 1

## Data Availability

All data generated or analyzed during this study are included in this published article and its Supplementary Information file (Dataset.xlsx).
